# Spatio-temporal variation of ecosystem services value in the Northern Tianshan Mountain Economic zone from 1980 to 2030

**DOI:** 10.7717/peerj.9582

**Published:** 2020-08-05

**Authors:** Lei Shi, Ümüt Halik, Zulpiya Mamat, Zhicheng Wei

**Affiliations:** 1 College of Resources and Environmental Science, Xinjiang University, Urumqi, China; 2 Ministry of Education Key Laboratory of Oasis Ecology, Xinjiang University, Urumqi, China

**Keywords:** Land use and land cover (LULC), Ecosystem services value (ESV), Sensitivity analysis, Northern Tianshan Mountain Economic Zone (NTMEZ)

## Abstract

Rapid agricultural land expansion and urbanization have accelerated land use and land cover changes (LUCC) in the Northern Tianshan Mountain Economic Zone and have significantly impacted on the ecosystem services (ESs). However, the spatiotemporal variations of ecosystem service value (ESV) to LUCC are not well understood. Based on the land use and land cover (LULC) data from 1980 to 2019, we used a CA-Markov model to predict LUCC in 2020 and 2030, assess the spatial-temporal changes of ESV and LULC during 1980–2030, and explore the elastic response of ESV to LUCC. We found that cropland and built-up land expanded rapidly by 34.38% and 196.66%, respectively between 1980 and 2030, while grassland and unutilized land decreased significantly by 11.45% and 10.26%, respectively. The ESV of water body, cropland, grassland and forestland accounts for more than 90% of the total ESV. Our research shows that the ESV of cropland increased 32 million yuan from 1980 to 2030, mainly due to the expansion of cropland area. However, the loss caused by the reduction of grassland area was 45 million yuan. Water conservation, waste treatment, soil formation and retention, and biodiversity conservation are the primary ecosystem service function, accounting for 71.82% of the total ESV. Despite notable increases in the ESV from 1980 to 2010, grassland degradation still remains a main ecological and environmental issue from 2010 to 2030. The results suggest that effective land use policies should be developed to control the expansion of croplands and protect water body, grassland and forestland to maintain more sustainable ESs.

## Introduction

Ecosystem service (ES) refers to the benefits directly or indirectly obtained by human through ecosystem structure, process and function, which includes four aspects: provisioning services, regulating services, supporting services and cultural services (Costanza et al., 1997). By evaluating the ecosystem service value (ESV), the benefits obtained from the ecosystem can be quantified to help decision makers make optimal decision on the rational allocation of resources, so as to realize the sustainable management of the ecosystem (Deal, Smith & Gates, 2017). With increasing importance of ES, the evaluation methods of ESV become more and more diversified. Talberth (2015) divided the evaluation methods into four categories, including stated preference method, revealed preference method, cost-based method and benefit transfer method (BTM). A series of models have also been developed to accurately evaluate ESV, including Integrated Valuation of Ecosystem Services and Trade-offs (InVEST) (Redhead et al., 2016), Social Values for Ecosystem Services (SolVES) (Sherrouse, Semmens & Clement, 2014; Zhou et al., 2020), and so on (Sun et al., 2019). In regional or global ESV assessment, BTM is simple and easy to operate and has been widely used. This approach potentially assumes that the value of each ecosystem service function (ESF) is derived from a single or a multiple case study utilizing the specific value of a particular land cover (Costanza et al., 1997). Recently, the evaluation has been updated based on more than 300 case studies worldwide (Costanza et al., 2014; De Groot et al., 2012). Costanza et al. (2014) claimed that the models can be applied at various scales. However, the spatial heterogeneity of ecosystem structure eventually leads to the spatial heterogeneity of ESF (Wilson & Howarth, 2002; Kindu et al., 2016). Since the limitations and uncertainties of land use and land cover changes (LUCC) are ignored, and simply using the global value coefficient to estimate the small-scale ESV limits the accuracy of the assessment (Zhang et al., 2020). Based on the results of Costanza et al. (2014) and Xie et al. (2010), it is an important issue to adjust the equivalent factor to a small scale and reflect the changing relationship between LUCC and ESV based on the social and economic status quo.

Land use and land cover changes can affect the supply and value of ESs by changing the structure, processes and functions of ecosystems (Daily, 1997). Overuse of land resources may lead to degradation of regional ecosystem and the loss of ESV. Recent studies have shown that with rapidly increasing population, land pressure is gradually increasing (Han, Song & Deng, 2016). Excessive land reclamation and urbanization leads to land degradation, loss of biodiversity, water shortage and carbon loss, and further leads to a significant decline in ESV (Collin & Melloul, 2001).

The Northern Tianshan Mountain Economic Zone (NTMEZ) is an important hub of the “Silk Road” from Asia to Europe, and the largest and most developed economic area in Xinjiang. However, the special geographical environment of the NTMEZ has resulted in limited water resources and a fragile ecosystem (Zhou, Zhao & Yang, 2017). Under the influence of long-term agricultural activities and the development and utilization of water and land resources, the ecological and environmental problems caused by land use in this oasis are becoming more and more prominent (Wang et al., 2016). Under China’s “One Belt One Road” strategy, the NTMEZ has entered an important stage of development. According to the development plan of urban agglomeration for the NTMEZ in Xinjiang, the urban agglomeration will build an urban system with Urumqi as the core area, which will accelerate population and industrial agglomeration. The rapid urbanization will directly lead to decrease of grassland and forestland, and destruction of ecosystem structure, function, and services in the NTMEZ. Therefore, it is urgent to evaluate the impacts of human disturbances on ES in NTMEZ. In addition to the oasis LUCC and urbanization, there has been no quantitative spatial and temporal assessment of ESV in response to LUCC in NTEMZ.

Based on the research gap identified above, this study quantitatively evaluates the LUCC and ESV of NTMEZ from 1980 to 2030. There are three specific objectives: (1) to reveal the spatial and temporal variations of the land use and land cove (LULC) and ESV from 1980 to 2030; (2) to evaluate changes in ESV in response to LUCC; and (3) to explore the elasticity of the response of ESV to LUCC by 50% adjustment of value coefficients. These results will provide information to government for the sustainable development of land use and ecological environment.

## Materials and Methods

### Study area

**Figure 1:**
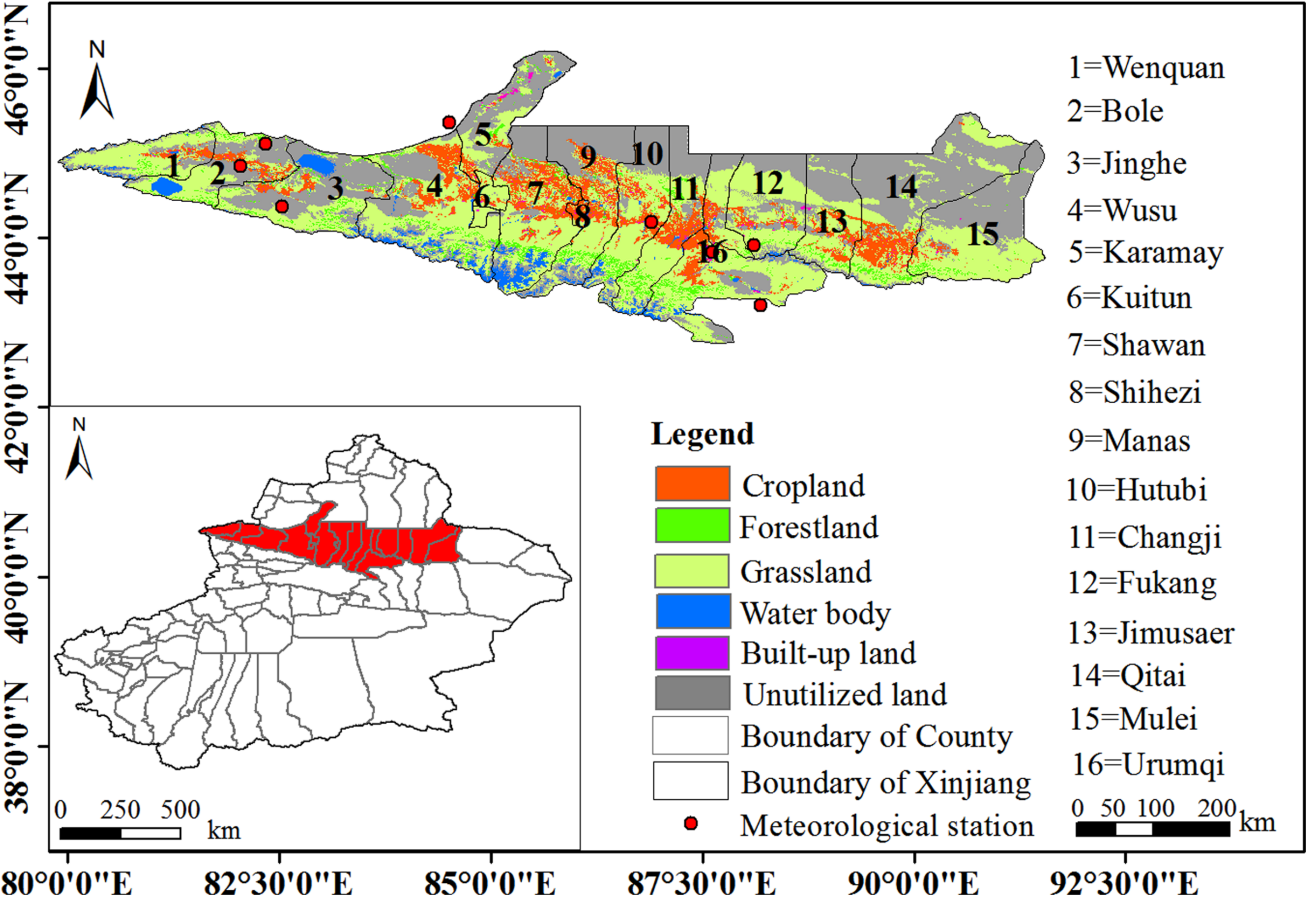
The location of the Northern Tianshan Mountain economic zone.

The NTMEZ is located in the north of the Tianshan Mountain and the south of the Junggar basin in Xinjiang, China (Fig. 1), covers an area of 9.54 × 10^4^ km^2^, accounting for 5.70% of Xinjiang (Xinjiang Bureau of Statistics, 2017), and is one of the 18 national key development areas. The population of the study area was 8.92 million in 2016 (Xinjiang Bureau of Statistics, 2017), accounting for 37.80% of the population of Xinjiang. Notably, the Gross Domestic Product (GDP) of the study area was 64.62 × 10^11^ yuan in 2016, accounting for 69.30% of the GDP of Xinjiang in that year (Xinjiang Bureau of Statistics, 2017). Among them, the yields of primary industry, secondary industry and tertiary industry were 6.22 × 10^11^ yuan (9.62%), 25.34 × 10^11^ yuan (39.21%) and 33.06 × 10^11^ yuan (51.17%), respectively. With the implementation of China’s “Western Development Strategy”, this region has become the most economically developed region in Xinjiang and an important hub of the “Silk Road Economic Belt”. The annual average temperature, average annual precipitation, and potential evaporation in the study area are 6.10–8.90 °C , 220 mm and 1,210 mm, respectively (Zhao et al., 2010). The evaporation of the study area is much higher than the precipitation, the climate belongs to the temperate arid continental climate, and the ecological environment is extremely fragile. With increase in human activity, the resource and environmental problems in this region have become increasingly prominent.

### Data preparation

The Landsat TM/ETM remote sensing image data of years: 1980, 1990, 2000, 2010, 2015 and 2019, were obtained from the U.S. Geological Survey (https://www.usgs.gov/) with a spatial resolution of 30 m. The radiometric correction, geometrical correction, and atmospheric correction of the images were done using the ERDAS 9.2 software. In this article, we referred to the ESV classification system presented by Xie et al. (2010). Meanwhile, considering the practical situation of the study area, supervised classification and visual interpretations were combined in order to classify the LULC types into six categories: cropland, forestland, grassland, water body, built-up land and unutilized land. The classification basis of LULC is shown in Table S1. There were more than 40 training sites for each LULC type for classification process, which were obtained using a GPS device; Google Earth was applied for validation of the LULC type (Elkhrachy, 2015). The confusion matrix method was used to evaluate the interpretation accuracy, and the Kappa coefficient was 87%. We also collected the digital elevation modal, meteorological data, the distribution data of the population and GDP, basic geographic information data, and grain yield data. Data descriptions and resources are shown in Table 1.

**Table 1:** Data descriptions and resource.

Layer name	Formats	Source
Landsat TM/ETM	Raster, 30 m	USA Geological Survey, https://www.usgs.gov/
Digital elevation	Raster, 30 m	International Scientific Data Network of Computer Network Information Center, http://www.gscloud.cn
Meteorological data	Point	China Meteorological Science Data Network, https://data.cma.cn/en
Population data	Raster, 500 m	Resources and Environmental Sciences Data Center, Chinese Academy of Sciences, http://www.resdc.cn
Gross domestic product data	Raster, 500 m	Resources and Environmental Sciences Data Center, Chinese Academy of Sciences, http://www.resdc.cn
Basic geographic information data	Shapefile	National Basic Geographic Information Center’s Database, http://ngcc.sbsm.gov.cn/
Grain yield data	Txt	Statistical Yearbook of Xinjiang from 1980 to 2015, http://www.xjtj.gov.cn/

### CA-Markov model

CA model is a lattice dynamic model with discrete time and space states, which focuses on the interaction between cells with different spatial and temporal characteristics (Chen et al., 2020). It has strong spatial computational simulation ability and is especially suitable for dynamic simulation and spatial display. The Markov model focuses on the quantitative prediction of LUCC, but it cannot carry out spatial expression and show the spatial distribution of LUCC (Wickramasuriya et al., 2009). However, CA can express the spatial-temporal dynamic evolution process of complex space system, which can make up the deficiency of the Markov model. By combining the CA model with Markov model, the CA-Markov model has the advantage of expressing the temporal and spatial patterns of LUCC, so as to better simulate the spatial and temporal patterns of future regional LUCC, and further provide support for the future land use and ecological environment sustainable development (Mitsova, Shuster & Wang, 2011).

The Markov chain was constructed based on the probability of change matrices for LUCC from *t* to *t*+1:

$${S_{\left( {t + 1} \right)}} = {S_{\left( t \right)}} \times A$$

where *S*_(t+1)_ is the state probability of any time, and *S*_(t)_ is the initial state probability. *A* is the transition probability matrix, and the formula is as follows:

$${\rm A} = \left[ {\matrix{ \; \cr {{A_{11}}\; {A_{12}} \ldots \; {A_{1n}}} \cr {{A_{21}}\; {A_{22}} \ldots \; {A_{2n}}} \cr { \ldots \; \; \; \ldots \; \; \ldots \; \; \ldots \; } \cr {{A_{n1}}\; {A_{n2}} \ldots \; {A_{nn}}} \cr } } \right]$$

where *A*_ij_ is the sum of areas from the ith to the jth land cover category from the initial to the forecast period, and *n* is the number of LULC categories. We accomplished this process by using the Markov model with the IDRISI software that integrates geographic information system and image processing functions.

The CA model was used to predict the spatial and temporal dynamic change pattern using a transition map of the LUCC (Yang, Zheng & Lv, 2012), and the model can be defined as follows (Wang, Zheng & Zang, 2012):

$${S_{\left( {t,t + 1} \right)}} = f\left( {{S_{\left( t \right)}},N} \right)$$

where *S* is a set of cellular states, *N* is the cellular field, *t* and *t*+1 represent different time periods, and *f* is the local transition rule of the cell.

To ensure the reliability of the simulation results, we used the kappa index to test the consistency level of the simulated and observed land cover maps (Mitsova, Shuster & Wang, 2011):

$${\rm Kappa} = \displaystyle{{{P_0} - {P_c}} \over {1 - {P_c}}}$$

where Kappa is the index of simulation accuracy, *P*_c_ is the expected simulation accuracy in a random state, and *P*_0_ is the actual simulation accuracy.

**Figure 2:**
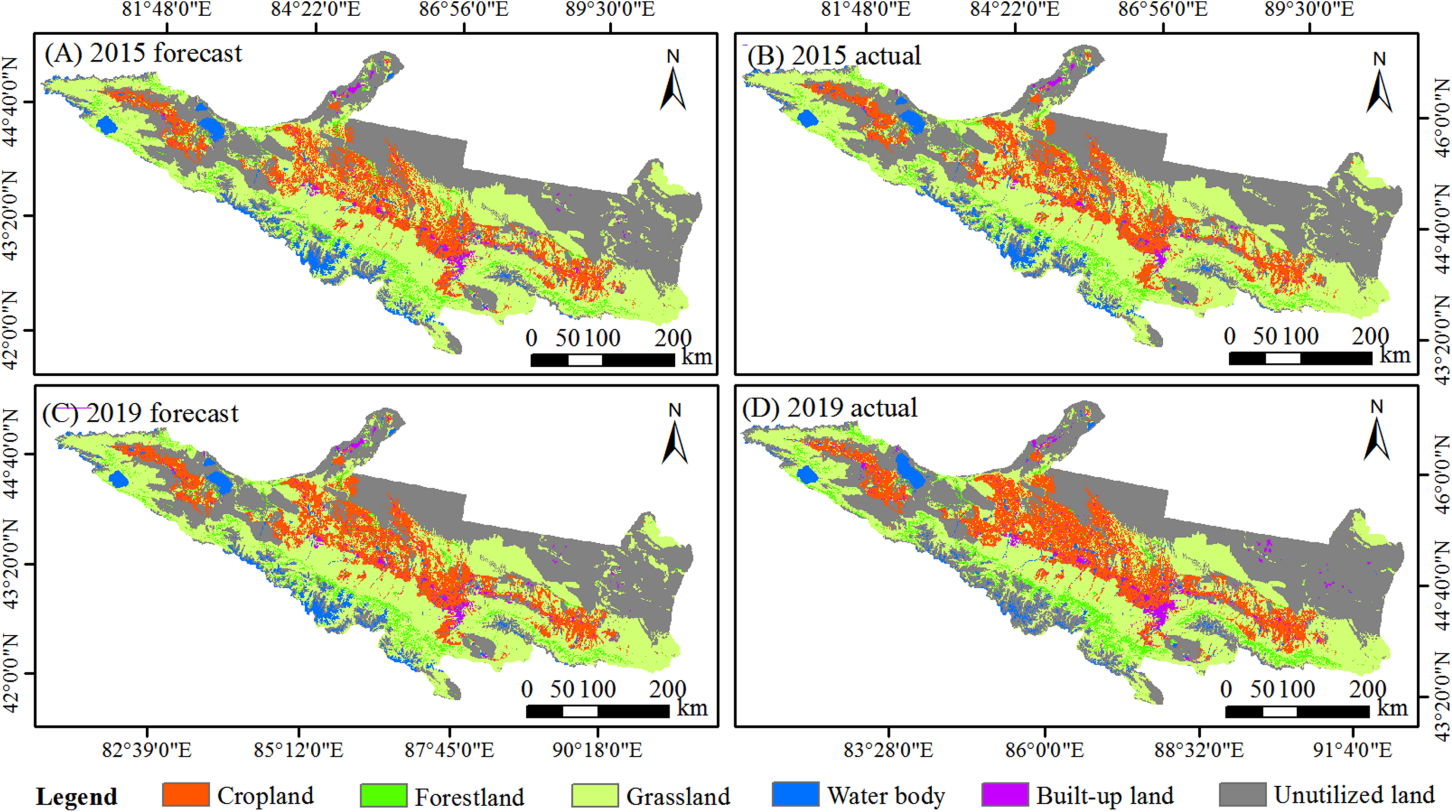
Comparison map of the actual and predicted land in 2015 and 2019. (A) Forecast LULC map in 2015. (B) Actual LULC distribution in 2015. (C) Forecast LULC map in 2019. (D) Actual LULC distribution in 2019.

In this study, the transition probability matrix from 2005 to 2010 was used to predict the LUCC from 2015 to 2019. The transition probability matrix is showed (Table S2). Then, the forecast data in 2015 and 2019 were compared with the respective actual data to verify the reliability of the CA-Markov model (Fig. 2). The Kappa coefficient was 0.79 and 0.72 in 2015 and 2019, respectively, which were above the required standard of 70%. The simulation results were accurate and reliable, can be used for prediction and simulation of future LUCC. Finally, the LUCC spatial data in 2020 and 2030 were predicted.

### Assessment of ESVs

In this article, the estimation coefficients of ESV were referred to Costanza et al. (1997) and Xie et al. (2010) were referred to. Given the spatial and temporal effects of ES, the ESV for LULC type was revised according to the actual conditions of the study area by consulting 30 ecologists. According to the specific conditions of the research area, the economic value of the annual natural grain yield per unit area of cropland was modified as follows: the average annual grain yield of the research area from 1990 to 2010 was used to replace the grain yield per unit area of each year; the average purchase price of grain (1.98 yuan/kg) in China in 2010 was used as the grain price for the research period (China Food Industry Association, 2010); the equivalent factor of the ESV of the research area was calculated as 1,304.86 yuan/hm^2^. Meanwhile, considering the negative impacts of built-up land on the ecosystem in terms of water supply and waste treatment, the replacement cost method was used to estimate the ESV of built-up land (Mayila et al., 2018). On this basis, the ESV coefficients of LULC in the study area were calculated (Table 2; Table S3). Notably, the equivalent value of ESs per standard unit is defined as 1/7 of the economic value of the average annual grain yield of 1 hm^2^ of cropland. The formula is given as follows:

$${E_a} = \displaystyle{1 \over 7}\mathop \sum \limits_{i = 1}^n \displaystyle{{{m_i} \times {p_i} \times {q_i}} \over M}\; {\rm (}{i} = 1,2,3 \ldots { n}{\rm )}$$

where *E*_a_ is the economic value of 1 hm^2^ farm’s annual grain crops (yuan/hm^2^); *P*_i_ is the national average price of *i* food crops (yuan/t); *q*_i_ represents the yield of grain crop *i* (t/hm^2^); *M*_i_ is the area of *i* food crops (hm^2^); *M* is the total area of food crops (hm^2^); *i* is the crop type, including rice, wheat and corn.

**Table 2:** ESV coefficients of LULC in the study area (CNY ha^1^yr^1^ ).

Ecosystem service	Forest land	Grass land	Crop land	Water body	Built-up land	Unutilized land
Gas regulation	4,567.01	1,043.89	652.43	0	0	0
Climate regulation	3,523.12	1,174.37	1,161.33	600.24	0	0
Water conservation	4,175.55	1,043.89	782.92	26,593.05	0	39.15
Waste treatment	1,709.37	1,709.37	2,139.97	23,722.36	−9,799.50	13.05
Soil formation and protection	5,088.95	2,544.48	1,905.10	13.05	−3,209.96	26.10
Biodiversity conservation	4,253.84	1,422.29	926.45	3,249.10	0	443.65
Food production	130.49	391.46	1,304.86	130.49	0	13.05
Raw material	3,392.64	65.24	130.49	13.05	0	0
Recreational culture	1,670.22	52.19	13.049	5,663.09	0	13.05
Total	28,511.19	9,447.19	9,016.58	59,984.41	−13,009.45	548.04

The calculation formula of the ESV is as follows:

$${\rm ESV} = \sum {A_i} \times \rm V{C_i}$$

$${\rm ES}{{\rm V}_f} = \sum {A_i} \times \rm V{C_{if}}$$

where *A*_i_ is the distribution area of land use type i in the research area (hm^2^); VC_i_ is the ESV coefficient (yuan·hm^−2^·a^−1^); ESV_f_ represents the functional value of individual f ecosystem services; VC_if_ represents the functional value of individual f ecosystem services of land use type i (yuan·hm^−2^·a^−1^).

### Elasticity for the response of ESV to LUCC

We used ESV coefficients of LULC to replace the six LULC categories that did not exactly match the report by Costanza et al. (1997) (Table S3), which resulted in uncertainties in the assessment of the ESV. Therefore, we used sensitivity analysis to evaluate the changes in ESV in response to 50% adjustments of the ESV coefficients for each LULC type (Mamat et al., 2019). The standard economic concept of elasticity was used to calculate the coefficient of sensitivity (CS) using the following formula:

$${\rm CS} = \displaystyle{{\left( {{\rm ESV}_{j} - {\rm ESV}_{i}} \right)/{\rm ESV}_{i}} \over {\left( {{\rm ESV}_{{jk}} - {\rm ESV}_{{ik}}} \right)/{\rm ESV}_{{ik}}}}$$

where ESV is the estimated total value of ecosystem services, VC is the value coefficient, and “*i*”, “*j*” and “*k*” represent the initial, adjusted values, and LULC categories, respectively. If CS > 1, then the estimated ESV is elastic with respect to that coefficient; if CS ≤ 1, the estimated ESV is inelastic. Thus, when CS < 1, even if the accuracy of VC values used as proxy biomes is low, the results of estimation of ESV are credible.

## Results

### Changes of LULC

**Figure 3:**
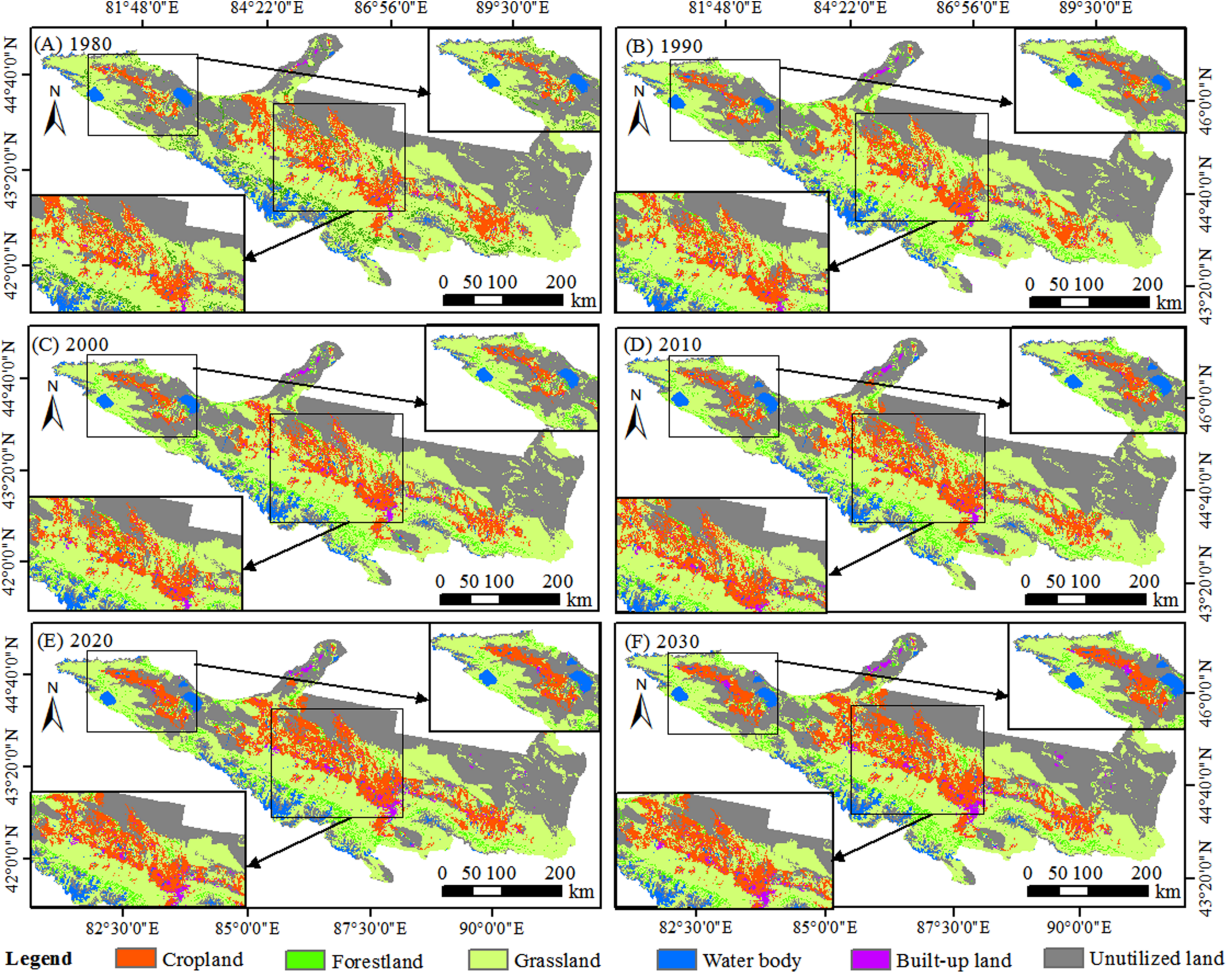
Spatial distribution of LULC in NTMEZ. (A) 1980. (B) 1990. (C) 2000. (D) 2010. (E) 2020. (F) 2030.

According to the simulation results of the CA-Markov model, the spatial distribution of LULC is showed in Fig. 3 and the dynamic change of land use area is showed in Table 3. In 1980, LULC was dominated by grassland and unutilized land, which accounted for 42.73% and 38.83% of the total area, respectively, followed by cropland (10.65%) and forestland (3.90%). During 1980–2010, cropland and built-up land increased substantially. The cropland expanded 1,562.31 km^2^ with an increase rate of 15.31% by 2010. With the increase of urbanization, built-up land also increase from 643.15 km^2^ in 1980 to 1,095.13 km^2^ in 2010, with an increase rate of 70.28%. Cropland and built-up land were expected to continue to increase during 2020–2030. However, the spatial distribution of unutilized land mainly concentrated in the Gurbantunggut Desert, which decreased from 37,206.00 km^2^ to 35,713.36 km^2^ during 1980–2010, and it will be further decrease to 33,390.00 km^2^ in 2030. The coverage of grassland mainly concentrated in the north slope of Tianshan Mountain, which decreased from 42.73% in 1980 to 32.83% in 2030 with the expansion of cropland.

**Table 3:** Area changes of LULC in NTMEZ from 1980 to 2030.

LULC		Forest land	Grass land	Crop land	Water body	Built-up land	Unutilized land
Area (km^2^)	1980	3,722.82	40,938.98	10,204.99	3,093.59	643.15	37,206.00
	1990	3,977.29	40,888.34	9,847.97	2,956.23	812.80	36,917.37
	2000	4,012.11	40,428.76	10,411.99	3,272.74	994.48	36,279.92
	2010	3,970.33	39,461.50	11,767.30	3,392.38	1,095.13	35,713.36
	2020	3,851.32	38,142.28	13,732.84	3,368.96	1,540.78	34,763.82
	2030	3,846.26	36,252.00	13,713.22	3,357.56	1,908.00	33,390.00
Change (%)	1980–2010	6.65	−3.61	15.31	9.66	70.28	−4.01
	2010–2030	−3.12	−8.13	16.54	−1.03	74.23	−6.51
	1980–2030	3.32	−11.45	34.38	8.53	196.66	−10.26

Due to water shortage in the arid areas, the water body only accounted for 3% of the total area. Water body increased from 3,093.59 km^2^ in 1980 to 3,392.38 km^2^ in 2010. However, with the increase of cropland area, water consumption will further increase, and water body will further decrease 1.03% during 2010–2030.

### Changes of ESVs

**Figure 4:**
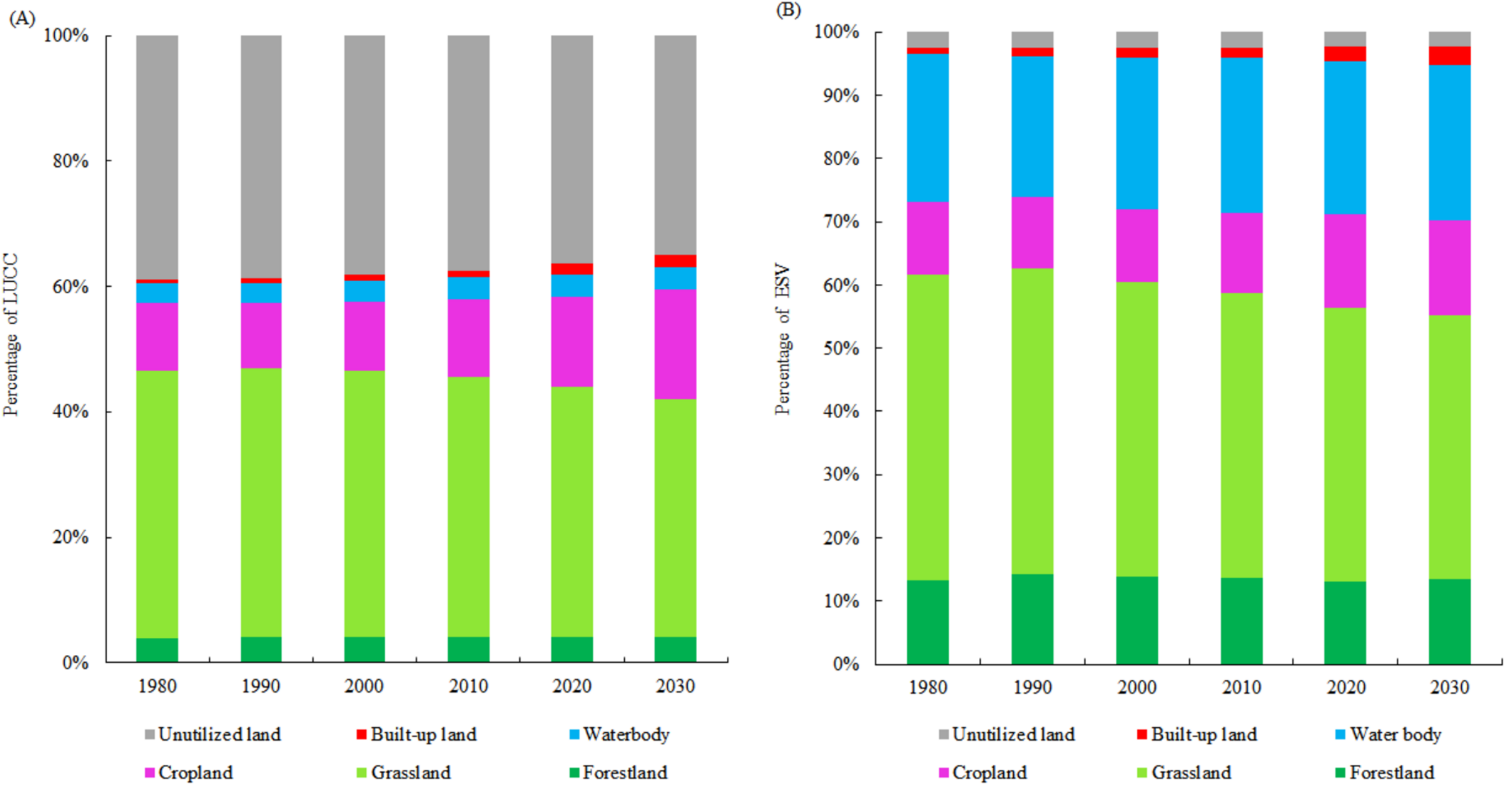
The percentage of LUCC and ESV of different land use types. (A) The percentage of LULC area and (B) The percentage of ESV of different land use types.

According to the ESV of different types of LULC per unit area (Table 2), we calculated the ESV of different LULC in NTMEZ from 1980 to 2030 (Table 4). The total ESV in NTMEZ was approximately 7.83 × 10^8^ yuan in 1980. Grassland had the highest contribution of 49.43%, followed by cropland, water body and forestland with a contribution of 23.75%, 13.54% and 11.75%, respectively. Due to LUCC, the ESV increased by 2.35% from 1980 to 2010. Cropland and water body increased and played an important role in improving ESV, which over compensated the ESV loss in grassland and built-up land (Fig. 4). Notably, with the acceleration of urbanization, grassland and unutilized land gradually decreased, and the ESV will decrease by 0.3 × 10^8^ yuan from 2010 to 2030.

**Table 4:** Changes of ESV for different LULC during 1980–2030.

LULC	ESV (×10^8^ yuan)						Change rate (%)		
	1980	1990	2000	2010	2020	2030	1980–2010	2010–2030	1980–2030
Forestland	1.06	1.13	1.14	1.13	1.10	1.10	6.65↑	−3.12↓	3.32↑
Grassland	3.87	3.86	3.82	3.73	3.60	3.42	−3.61↓	−8.13↓	−11.45↓
Cropland	0.92	0.89	0.94	1.06	1.24	1.24	15.31↑	16.54↑	34.38↑
Water body	1.86	1.77	1.96	2.03	2.02	2.01	9.66↑	−1.03↓	8.53↑
Built-up land	−0.08	−0.11	−0.13	−0.14	−0.20	−0.25	−70.28↓	−74.23↓	−196.66↓
Unutilized land	0.20	0.20	0.20	0.20	0.19	0.18	−4.01↓	−6.51↓	−10.26↓
Total	7.83	7.75	7.93	8.01	7.95	7.71	2.35↑	−3.78↓	−1.51↓

**Figure 5:**
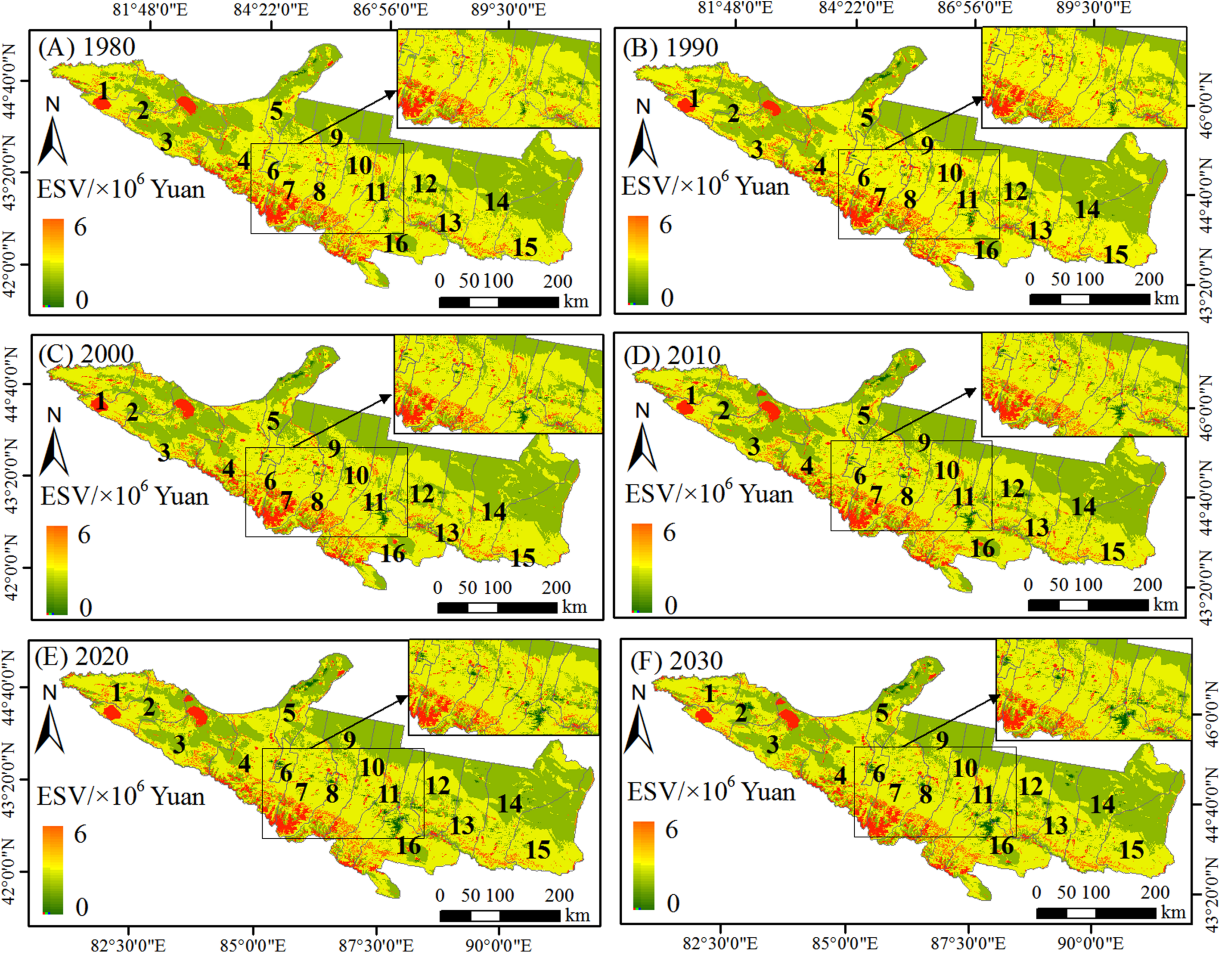
Ecosystem service value change from 1980 to 2030. (A) 1980. (B) 1990. (C) 2000. (D) 2010. (E) 2020. (F) 2030. Notes: 1 = Wenquan, 2 = Bole, 3 = Jinghe, 4 = Wusu, 5 = Karamay, 6 = Kuitun, 7 = Shawan, 8 = Shihezi, 9 = Manas, 10 = Hutubi, 11 = Changji, 12 = Fukang, 13 = Jimusaer, 14 = Qitai, 15 = Mulei, 16 = Urumqi.

The spatial distribution of the ESV in the study area was unbalanced. The high ESV area was mainly distributed in Aibi Lake Basin, Selimu Lake, Baiyang River, Toutun River and Manas River Basin (Fig. 5). In addition to the higher ESV distribution along river water, the northern part of the Tianshan Mountain also had a higher distribution of ESV. The main distribution of the lowest ESV areas was located in the edge of the Gurbantunggut Desert. Notably, compared with the period from 1980 to 2010, the decrease of ESV between 2010 and 2030 shifted from Shihezi, Hutubi, Changji and Jimusaer to Karamay, Fukang, Urumqi and Manas (Table 5). With the urban population growth and industrial agglomeration, the ecological environment will gradually deteriorate during 2010–2030.

**Table 5:** Ecosystem service value change of different regions.

LULC	ESV (×10^8^ yuan)						Change rate (%)		
	1980	1990	2000	2010	2020	2030	1980–2010	2010–2030	1980–2030
Wenquan	0.49	0.50	0.53	0.53	0.56	0.54	8.60↑	0.78↑	9.44↑
Bole	0.53	0.52	0.48	0.56	0.59	0.56	4.61↑	0.93↑	5.59↑
Jinghe	0.84	0.75	0.85	0.86	0.89	0.89	2.78↑	3.48↑	6.36↑
Wusu	1.18	1.19	1.17	1.19	1.23	1.22	0.36↑	2.66↑	3.03↑
Karamay	0.26	0.25	0.28	0.27	0.26	0.24	5.92↑	−13.61↓	−8.50↓
Kuitun	0.04	0.04	0.05	0.04	0.04	0.04	0.50↑	−4.25↓	−3.77↓
Shawan	0.85	0.86	0.86	0.86	0.87	0.84	1.49↑	−2.77↓	−1.33↓
Shihezi	0.04	0.05	0.04	0.04	0.04	0.03	−17.48↓	−5.38↓	−21.92↓
Manas	0.74	0.72	0.73	0.75	0.72	0.67	0.99↑	−10.21↓	−9.32↓
Hutubi	0.54	0.58	0.42	0.52	0.41	0.42	−2.85↓	−19.81↓	−22.10↓
Changji	0.51	0.55	0.50	0.49	0.52	0.52	−3.43↓	4.57↑	0.99↑
Fukang	0.24	0.23	0.37	0.25	0.18	0.16	5.95↑	−37.76↓	−34.05↓
Jimusaer	0.25	0.24	0.25	0.24	0.25	0.25	−0.42↓	1.70↑	1.27↑
Qitai	0.36	0.39	0.39	0.40	0.40	0.38	9.14↑	−3.64↓	5.17↑
Mulei	0.27	0.28	0.30	0.30	0.30	0.28	9.02↑	−4.80↓	3.79↑
Urumqi	0.68	0.60	0.70	0.70	0.68	0.67	2.88↑	−4.28↓	−1.52↓
Total	7.83	7.75	7.93	8.01	7.95	7.71	2.35↑	−3.78↓	−1.51↓

### Changes in values of ESF

**Figure 6:**
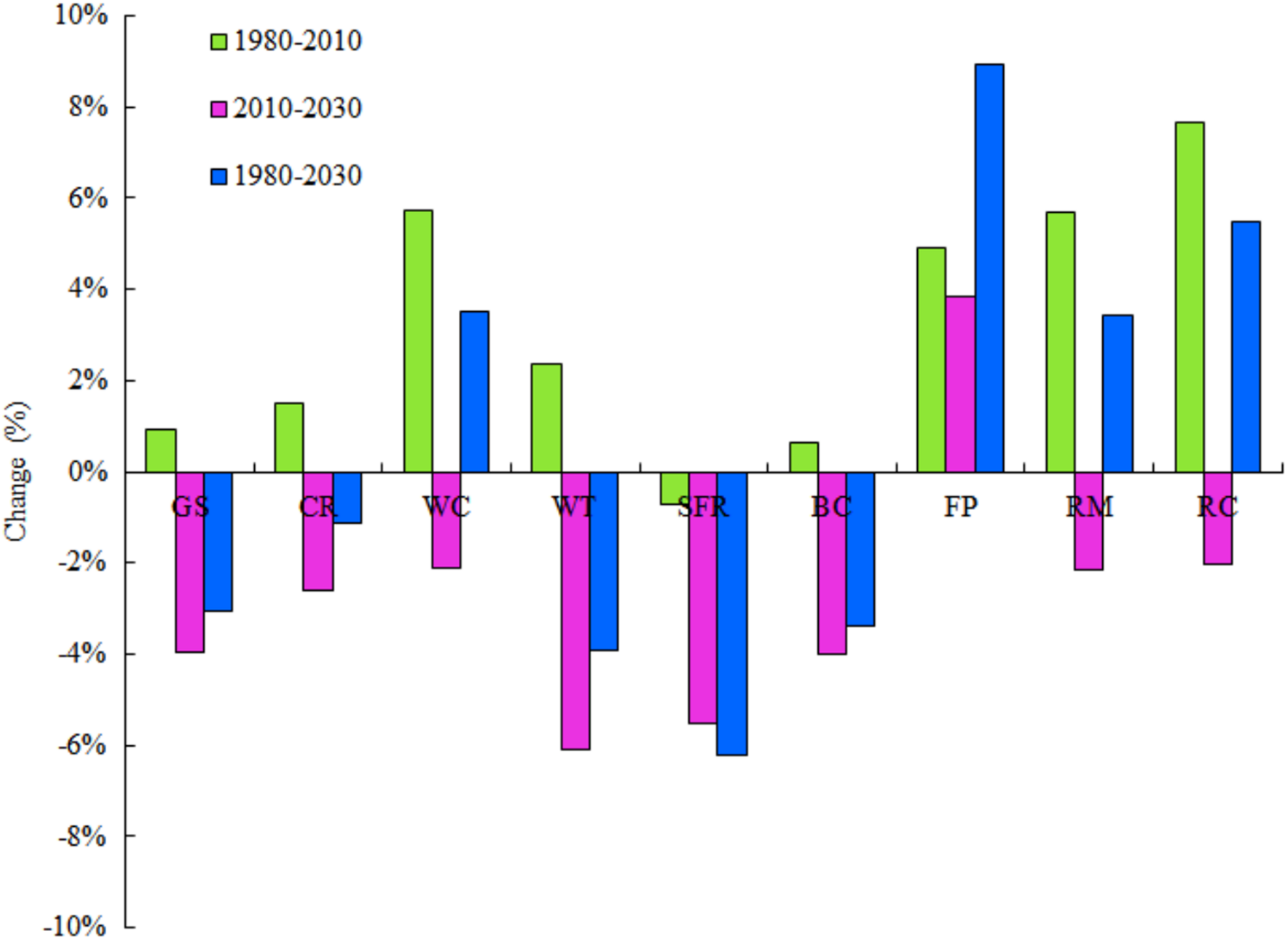
Change rate of ecosystem service function in NTMEZ from 1980 to 2030. GS, gas regulation, CR, climate regulation, WC, water conservation, WT, waste treatment, SFR, soil formation and retention, BC, biodiversity conservation, FP, food production, RM, raw material, RC, recreational culture.

The contribution and change of individual ESF are summarized in Table 6. Water conservation, waste treatment, soil formation and protection, and biodiversity conservation contributed the most to the ESV, with a contribution of 20.14%, 20.67%, 17.22% and 13.80% in 2030. Most of individual ESFs increased except food production during 1980–2010 with cropland expansion. Water conservation, food production, raw material and recreational culture increased by 5.73%, 4.92%, 5.70% and 7.66%, respectively (Fig. 6). However, most of individual ESFs were projected to decrease from 2010 to 2030. Due to the increase of cropland area, only the ESV of food production will increase during 2010–2030.

**Table 6:** Changes of ESV components in the study area during 1980–2030.

Ecosystems service	ESV (×10^8^ yuan)						Change rate (%)		
	1980	1990	2000	2010	2020	2030	1980–2010	2010–2030	1980–2030
Gas regulation	0.66	0.67	0.67	0.67	0.66	0.64	0.01↑	−0.03↓	−0.02↓
Climate regulation	0.75	0.75	0.76	0.76	0.76	0.74	0.01↑	−0.02↓	−0.01↓
Water conservation	1.50	1.47	1.56	1.59	1.58	1.55	0.09↑	−0.04↓	0.05↑
Waste treatment	1.66	1.60	1.67	1.70	1.66	1.59	0.04↑	−0.11↓	−0.07
Soil formation and retention	1.42	1.41	1.41	1.41	1.39	1.33	−0.01↓	−0.08↓	−0.09↓
Biodiversity conservation	1.10	1.10	1.11	1.11	1.10	1.06	0.01↑	−0.05↓	−0.04↓
Food production	0.31	0.30	0.31	0.32	0.34	0.34	0.01↑	0.02↑	0.03↑
Raw material	0.17	0.18	0.18	0.18	0.17	0.17	0.01↑	−0.01↓	0.00
Recreational culture	0.27	0.26	0.28	0.29	0.28	0.28	0.02↑	−0.01↓	0.01↑
Total	7.83	7.76	7.94	8.01	7.95	7.71	0.18↑	−0.30↓	−0.12↓

### Ecosystem sensitivity analysis

In this article, the value index of LULC type was adjusted by 50% to measure the change of total ESV (Table 7). The results showed that the sensitivity index of ESV coefficient of all LULC types was less than 1, and the ESV of LULC was inelastic to VC, indicating that the ESV coefficient had little impact on the change of ESV. The sensitivity index of unutilized land was the smallest and the sensitivity index of grassland was the largest, indicating that grassland contributed the most to ESV. In addition, from the change of the sensitivity index in different periods, the CS value increased significantly due to the large increase of the cropland area, while other sensitivity index did not change significantly. The sensitivity index of unutilized land and built-up land was close to zero, and the VC change of the two land types had little impact on the change of ESV in NTMEZ. Overall, this study constructed the ESV coefficient suitable for the local actual situation.

**Table 7:** Percentage change in estimated total ESV and coefficient of sensitivity.

Change of coefficient	1980		1990		2000		2010		2020		2030	
	%	CS	%	CS	%	CS	%	CS	%	CS	%	CS
Forestland VC ± 50%	0.90	0.02	0.90	0.02	0.90	0.02	0.89	0.02	0.89	0.02	0.87	0.02
Grassland VC ± 50%	30.93	0.62	29.69	0.60	29.52	0.60	29.51	0.60	29.48	0.60	29.27	0.60
Cropland VC ± 50%	15.27	0.30	15.27	0.30	17.59	0.35	18.01	0.36	18.38	0.37	18.41	0.37
Water body VC ± 50%	2.13	0.04	2.02	0.04	2.73	0.06	2.85	0.06	2.41	0.05	2.15	0.04
Built-up land VC ± 50%	0.07	0.00	0.14	0.00	0.22	0.00	0.22	0.00	0.27	0.01	0.29	0.01
Unutilized land VC ± 50%	0.00	0.00	0.00	0.00	0.00	0.00	0.00	0.00	0.00	0.00	0.00	0.00

## Discussion

### Impact of LUCC change on ESV

Due to the acceleration of urbanization, population growth and agricultural intensification globally, the LULC is changing rapidly, which may lead to changes in ES (Nahuelhual et al., 2014). These effects are particularly significant in arid regions. Zhang et al. (2020) shows that the population showed a sharp increase in NTMEZ from 1980 to 2015, especially during the “Western Development” period, which led to a sharp increase in the population in 2001. Population growth led to the overexploitation of water body, cropland and built-up land to meet demands of water, food, energy and land (Granit et al., 2012). This study found rapid expansion of cropland (34.38%) and built-up land (196.66%), and shrinking of grassland (11.45%) and unutilized land (10.26%) during 1980–2030. Correspondingly, the ESV of cropland increased 0.32 × 10^8^ yuan from 1980 to 2030, which was mainly caused by the expansion of cropland area. However, the area of grassland decreased sharply during 1980–2030, causing a loss of 0.45 × 10^8^ yuan.

Increase in cropland may yield short-term economic benefits, but large increases in cropland may result in loss of ES (Li et al., 2019). Although short-term economic benefits can be gained from expanding agricultural production to meet the needs of population growth, it also reduces the ESV of grassland and unutilized land (Coupe, Barlow & Capel, 2012). In this study, we found the increase of cropland has led to increase in ESV of raw material by 9.12% from 0.307 × 10^8^ yuan to 0.335 × 10^8^ yuan and food production by 3.00 % from 0.167 × 10^8^ yuan to 0.172 × 10^8^ yuan in NTMEZ during 1980–2030. However, the ESV of soil formation and retention, biodiversity conservation and gas regulation were projected to decrease. These findings are consistent with many previous studies around the world (Sutton et al., 2016; Qiao et al., 2019). Agriculture and urban sprawl have a negative impact on the provision of ESFs, especially in Shihezi, Hutubi and Fukang. Our results showed that the ESV in Shihezi, Hutubi and Fukang, decreased by more than 20% from 1980 to 2030, which was mainly caused by the increase of cropland area and shrinkage of forestland and grassland area. Therefore, a series of protection measures should be carried out urgently. We suggest that a “Red Line for Ecological Protection” should be drawn and ecological land should be integrated into the land use classification system in order to restrict the encroachment of cropland on ecological land.

### Limitations and future work

Remote sensing images are the most important data source in our ESV evaluation. However, during the data processing, the spatial and temporal resolution, spectral resolution and classification method of LULC data will affect the interpretation accuracy. ESV is inevitably influenced by errors in the process of quantifying LULC data (Sexton et al., 2015). In this study, a land with more than 5% covered by plants is classified as grassland, which includes shrub pastures and mixed pastures with shrub canopy coverage less than 10% (Table S1). This significantly overstates the grassland area, leading to an overestimation of ESV. To address these limitations, higher spatial resolution remote sensing data and more accurate LULC classifications will be used to further accurately assess ESV.

Some ecosystem service costs are ignored when evaluating the ESV, such as soil salinization (Schäfer et al., 2012), loss of genetic resources (Leroy et al., 2018), and eutrophication of water (Wen & Théau, 2020), so our evaluation results may overestimate the ESV. For example, Cao et al. (2018) found that the ESV in China decreased by 52.66% in 2014 by considering such factors as water resource cost, ecological protection investment and land management. In the future research, we need to consider the multi-dimensional ES category to improve the evaluation accuracy by combining with the current situation of ecological environment in the research area.

In this study, the CA-Markov model was used to predict the spatial pattern of the ESV, and the reliability of the model was verified. The advantages of using the CA-Markov model in predicting large-scale ESV were demonstrated. However, the study is based on the assumption that the land use policy is basically unchanged (Zhang et al., 2020), and the natural and human driving factors of LUCC are selected based on the existing research results and experience (López-Marrero et al., 2011). The lack of quantitative analysis of the assumed conditions and the influence of various factors may cause certain e rrors in the prediction results (Kindu et al., 2016; Cao et al., 2018).

The simple benefit transfer approach used in this article to estimate the ESV has some limitations (Schulp et al., 2014). This equivalent factor method is only a static evaluation method, which lacks consideration of spatial and temporal differences of ecosystem types and quality, and the estimation results are insufficient to reflect the dynamic changes of ESFs in spatial and temporal scale, which limits the accuracy of the evaluation (Schmidt, Manceur & Seppelt, 2016; Cao et al., 2018). For example, in the study, we assumed that the cropland ESV had homogeneity in the whole region, and extended the unit value of one region to all regions. In fact, cropland with different crop growing structures provides different ecosystem services functions (Zheng, Zhang & Cao, 2018). In a recent study, a series of ES evaluation models were used to reveal the relationship between land use structure and ecosystem, including InVEST (Ouyang et al., 2016), SolVES (Sherrouse, Semmens & Clement, 2014; Zhou et al., 2020), and Coupling Coordination Model (Sun et al., 2019). For example, Ouyang et al. (2016) quantitatively evaluated the types of ESV in China based on the InVEST model and LUCC, revealing the imbalance between supply and demand. Zhang et al. (2017) used the coupling coordination model to evaluate the coordination degree of LUCC and ESV in Beijing from 2006 to 2015. These models can accurately simulate the dynamic changes of ES such as carbon storage, water and soil conservation, and cultural services in terrestrial ecosystems according to the spatial and temporal changes of LULC (Leh et al., 2013; Zhou et al., 2020; Wen & Théau, 2020). Due to the large amount of data and diverse types of data needed for ESF assessment models, the difficulty of data acquisition limits the application of these models in arid areas (Zhang, Yushanjiang & Jing, 2018). Therefore, there are uncertainties in our assessment results. In the future, we will find more advanced approach to integrate different data sources to improve the accuracy in estimating ESV.

## Conclusions

The study results showed that the cropland ESV increased about 14 million yuan during 1980–2010. This increase was mainly attribute to the increase of cropland area (15.31%). However, between 1980 and 2030, the grassland area decreased sharply by 3.61%, resulting in a loss of 14 million yuan. It must be pointed out that changes in cropland may yield short-term economic benefits but unsustainable agricultural production and rapid urbanization may lead to loss of natural ecosystem services. This trend will continue to intensify in the future, and ESV will continue to gradually decrease 30 million yuan with the urban population growth and industrial agglomeration from 2010 to 2030. Ecological protection red lines should be made to control farmland expansion and protect water bodies, grassland and forestland for more sustainable ecosystem services.

## Supplemental Information

10.7717/peerj.9582/supp-1 Supplemental Information 1 LULC categorical data.

10.7717/peerj.9582/supp-2 Supplemental Information 2 The raw transfer matrix data from IDRISI 17.0.

10.7717/peerj.9582/supp-3 Supplemental Information 3 Value comparison of ecosystem services between Costanza (1997) and this study (CNYha1yr1 ).

10.7717/peerj.9582/supp-4 Supplemental Information 4 Meteorological station information.

10.7717/peerj.9582/supp-5 Supplemental Information 5 Raw Data.

## Additional Information and Declarations

### Data Availability

The following information was supplied regarding data availability: Raw data are available in the Supplemental Files.

## References

[ref-2] Cao S, Yu Z, Zhang J, Feng F, Xu D, Mu X (2018). Cost–benefit analysis of ecosystem services in China. Ecological Engineering.

[ref-3] Chen S, Feng Y, Tong X, Liu S, Xie H, Gao C, Lei Z (2020). Modeling ESV losses caused by urban expansion using cellular automata and geographically weighted regression. Science of the Total Environment.

[ref-4] China Food Industry Association (2010). China food development report. http://www.cnfia.cn/.

[ref-5] Collin ML, Melloul AJ (2001). Combined land-use and environmental factors for sustainable groundwater management. Urban Water.

[ref-6] Costanza R, De Groot R, Sutton P, Van der Ploeg S, Anderson SJ, Kubiszewski I, Farber S, Turner RK (2014). Changes in the global value of ecosystem services. Global Environmental Change.

[ref-7] Costanza R, D’Arge R, Naeem S, O’Neil RV, Paruelo J, Raskin RG, Sutton P, Van den Belt M (1997). The value of the world’s ecosystem services and natural capital. Nature.

[ref-8] Coupe RH, Barlow JRB, Capel PD (2012). Complexity of human and ecosystem interactions in an agricultural landscape. Environmental Development.

[ref-9] Daily GC (1997). Nature’s service: societal dependence on natural ecosystems.

[ref-10] De Groot R, Brander L, Van der Ploeg S, Costanza R, Bernard F, Braat L, Christie M, Crossman N, Ghermandi A, Hein L, Hussain S, Kumar P, McVittie A, Portela R, Rodriguez LC, Ten Brink P, Van Beukering P (2012). Global estimates of the value of ecosystems and their services in monetary units. Ecosystem Services.

[ref-11] Deal RL, Smith N, Gates J (2017). Ecosystem services to enhance sustainable forest management in the US: moving from forest service national programmes to local projects in the Pacific Northwest. Forestry: An International Journal of Forest Research.

[ref-12] Elkhrachy I (2015). Flash flood hazard mapping using satellite image and GIS tools: a case study of Najran city, Kingdom of Saudi Arabia (KSA). International Journal of Remote Sensing.

[ref-13] Granit J, Jägerskog A, Lindström A, Björklund G, Bullock A, Löfgren R, De Gooijer G, Pettigrew S (2012). Regional options for addressing the water, energy and food nexus in central Asia and the Aral Sea Basin. International Journal of Water Resources Development.

[ref-14] Han Z, Song W, Deng XZ (2016). Responses of ecosystem service to land use change in Qinghai province. Energies.

[ref-15] Kindu M, Schneider T, Teketay D, Knoke T (2016). Changes of ecosystem service values in response to land use/land cover dynamics in Munessa–Shashemene landscape of the Ethiopian highlands. Science of the Total Environment.

[ref-16] Leh M, Matlock M, Cummings E, Thoma G, Cothren J (2013). Measuring ecosystem service change: a case study from a northwest Arkansas dairy farm. International Dairy Journal.

[ref-17] Leroy G, Baumung R, Boettcher P, Besbes B, From T, Hoffmann I (2018). Animal genetic resources diversity and ecosystem services. Global Food Security.

[ref-18] Li J, Chen H, Zhang C, Pan T (2019). Variations in ecosystem service value in response to land use/land cover changes in Central Asia from 1995–2035. PeerJ.

[ref-19] López-Marrero T, González-Toro A, Heartsill-Scalley T, Hermansen Báez LA (2011). Multi-criteria evaluation and geographic information systems for land-use planning and decision making.

[ref-20] Mamat Z, Halik U, Aishan T, Aini A (2019). Ecological effect of the riparian ecosystem in the lower reaches of the Tarim River in northwest China. PLOS ONE.

[ref-21] Mayila R, Mamat S, Nigela T, Yikiliman A, Ma C, Yierxiati A (2018). The ecosystem service value spatial-temporal changes in the Ugan-kuqa River Delta Oasis based on RS and GIS. Acta Ecologica Sinica.

[ref-22] Mitsova D, Shuster W, Wang X (2011). A cellular automata model of land cover change to integrate urban growth with open space conservation. Landscape and Urban Planning.

[ref-23] Nahuelhual L, Carmona A, Aguayo M, Echeverria C (2014). Land use change and ecosystem services provision: a case study of recreation and ecotourism opportunities in southern Chile. Landscape Ecology.

[ref-24] Ouyang ZY, Zheng H, Xiao Y, Polasky S, Liu J, Xu W, Wang Q, Zhang L, Xiao Y, Rao EM, Jiang L, Lu F, Wang XK, Yang GB, Gong SH, Wu BF, Zeng Y, Yang W, Daily GC (2016). Improvements in ecosystem services from investments in natural capital. Science.

[ref-25] Qiao X, Gu Y, Zou C, Xu D, Wang L, Ye X, Yang Y, Huang X (2019). Temporal variation and spatial scale dependency of the trade-offs and synergies among multiple ecosystem services in the Taihu Lake Basin of China. Science of the Total Environment.

[ref-26] Redhead JW, Stratford C, Sharps K, Jones L, Ziv G, Clarke D, Oliver TH, Bullock JM (2016). Empirical validation of the InVEST water yield ecosystem service model at a national scale. Science of the Total Environment.

[ref-27] Schmidt S, Manceur AM, Seppelt R (2016). Uncertainty of monetary valued ecosystem services—value transfer functions for global mapping. PLOS ONE.

[ref-28] Schulp CJE, Burkhard B, Maes J, Van Vliet J, Verburg PH (2014). Uncertainties in ecosystem service maps: a comparison on the European scale. PLOS ONE.

[ref-29] Schäfer RB, Bundschuh M, Rouch DA, Szöcs E, Von der Ohe PC, Pettigrove V, Kefford BJ (2012). Effects of pesticide toxicity, salinity and other environmental variables on selected ecosystem functions in streams and the relevance for ecosystem services. Science of the Total Environment.

[ref-30] Sexton JO, Noojipady P, Anand A, Song X-P, McMahon S, Huang C, Feng M, Channan S, Townshend JR (2015). A model for the propagation of uncertainty from continuous estimates of tree cover to categorical forest cover and change. Remote Sensing of Environment.

[ref-31] Sherrouse BC, Semmens DJ, Clement JM (2014). An application of social values for ecosystem services (SolVES) to three national forests in Colorado and Wyoming. Ecological Indicators.

[ref-32] Sun Y, Liu S, Dong Y, An Y, Sh F, Dong S, Liu G (2019). Spatio-temporal evolution scenarios and the coupling analysis of ecosystem services with land use change in China. Science of the Total Environment.

[ref-33] Sutton PC, Anderson SJ, Costanza R, Kubiszewski I (2016). The ecological economics of land degradation: Impacts on ecosystem service values. Ecological Economics.

[ref-34] Talberth J (2015). Valuing ecosystem services in the lower mekong basin: country report for Cambodia.

[ref-35] Wang RJ, Liu Y, Wang R, Song M (2016). Analysis of a river fed by precipitation in north slopes of the Tianshan Mountains: runoff characteristics and influence factors. Journal of Glaciology and Geocryology.

[ref-36] Wang SQ, Zheng XQ, Zang XB (2012). Accuracy assessments of land use change simulation based on Markov-cellular automata model. Procedia Environmental Sciences.

[ref-37] Wen X, Théau J (2020). Spatiotemporal analysis of water-related ecosystem services under ecological restoration scenarios: a case study in northern Shaanxi, China. Science of the Total Environment.

[ref-38] Wickramasuriya RC, Bregt AK, Van Delden H, Hagen-Zanker A (2009). The dynamics of shifting cultivation captured in an extended constrained celluar automata land use model. Ecological Modelling.

[ref-39] Wilson MA, Howarth RB (2002). Discourse-based valuation of ecosystem services: establishing fair outcomes through group deliberation. Ecological Economics.

[ref-40] Xie GD, Zhen L, Lu CX, Xiao Y, Li WH (2010). Applying value transfer method for ecoservice valuation in China. Journal of Natural Resources.

[ref-41] Xinjiang Bureau of Statistics (2017). Xinjiang statistical yearbook.

[ref-42] Yang X, Zheng XQ, Lv LN (2012). A spatiotemporal model of land use change based on ant colony optimization, Markov chain and cellular automata. Ecological Modelling.

[ref-43] Zhang D, Huang Q, He C, Wu J (2017). Impacts of urban expansion on ecosystem services in the Beijing–Tianjin–Hebei urban agglomeration, China: a scenario analysis based on the shared socioeconomic pathways. Resourse, Conserve Recycle.

[ref-45] Zhang Z, Xia F, Yang D, Huo J, Wang G, Chen H (2020). Spatiotemporal characteristics in ecosystem service value and its interaction with human activities in Xinjiang, China. Ecological Indicators.

[ref-46] Zhang F, Yushanjiang A, Jing Y (2018). Assessing and predicting changes of the ecosystem service values based on land use/cover change in Ebinur lake wetland national nature reserve, Xinjiang, China. Science of the Total Environment.

[ref-47] Zhao WY, Chen YN, Li JL, Jia GS (2010). Periodicity of plant yield and its response to precipitation in the steppe desert of the Tianshan Mountains region. Journal of Arid Environments.

[ref-48] Zheng X, Zhang J, Cao S (2018). Net value of grassland ecosystem services in mainland China. Land Use Policy.

[ref-49] Zhou L, Guan D, Huang X, Yuan X, Zhang M (2020). Evaluation of the cultural ecosystem services of wetland park. Ecological Indicators.

[ref-50] Zhou C, Zhao CX, Yang ZP (2017). Strategies for environmentally friendly development in the Northern Tianshan Mountain Economic Zone based on scenario analysis. Journal of Cleaner Production.

