# Supplementary material for: Spatio-temporal variation of ecosystem services value in the Northern Tianshan Mountain Economic zone from 1980 to 2030
Source: PeerJ. 2020 Aug 5;8:e9582. doi: 10.7717/peerj.9582 (PMC7414770; doi:10.7717/peerj.9582)
Supplement: Supplemental Information 1 [file peerj-08-9582-s001.docx]

**Table S1 LULC categorical data**

| LULC | Description |
| --- | --- |
| Cropland | Cultivated land for crops. Including long-term cultivated land, newly cultivated land, fallow, shifting cultivated land; intercropping land such as crop-fruit, crop-mulberry, and crop‐forestry land in which a crop is a dominant species; Bottomland and beach that has been cultivated for at least 3 years. |
| Forestland | Land where trees are grown, including arbor, shrub, bamboo, and for forestry use. |
| Grassland | Land covered by herbaceous plants with coverage greater than 5%, including shrub rangeland and mixed rangeland with the coverage of shrub canopies less than 10%. |
| Water body | Natural surface, natural water bodies, or constructed reservoirs for irrigation and water reservation. |
| Built-up land | Land used for urban and rural settlements, factories, and transportation facilities. |
| Unutilized land | Land that is not put into practical use or that is difficult to use. |
