# Supplementary material for: Spatio-temporal variation of ecosystem services value in the Northern Tianshan Mountain Economic zone from 1980 to 2030
Source: PeerJ. 2020 Aug 5;8:e9582. doi: 10.7717/peerj.9582 (PMC7414770; doi:10.7717/peerj.9582)
Supplement: Supplemental Information 2 [file peerj-08-9582-s002.docx]

**Table S2 The raw transfer matrix data from IDRISI 17.0**

| LULC | Cropland | Forestland | Grassland | Water body | Built-up land | Unutilized land |
| --- | --- | --- | --- | --- | --- | --- |
| Cropland | 0.87 | 0.02 | 0.06 | 0 | 0.02 | 0.01 |
| Forestland | 0.05 | 0.85 | 0.07 | 0.01 | 0 | 0.01 |
| Grassland | 0.15 | 0 | 0.8 | 0 | 0 | 0 |
| Water body | 0 | 0 | 0.01 | 0.73 | 0 | 0.2 |
| Built-up land | 0.02 | 0.02 | 0.02 | 0.02 | 0.9 | 0.02 |
| Unutilized land | 0.01 | 0 | 0.1 | 0 | 0 | 0.85 |
