# Supplementary material for: Spatio-temporal variation of ecosystem services value in the Northern Tianshan Mountain Economic zone from 1980 to 2030
Source: PeerJ. 2020 Aug 5;8:e9582. doi: 10.7717/peerj.9582 (PMC7414770; doi:10.7717/peerj.9582)
Supplement: Supplemental Information 3 [file peerj-08-9582-s003.docx]

**Table S3 Value comparison of ecosystem services between Costanza (1997) and this study. (CNYha1yr1 ).**

| Ecosystem service | Cropland | | Forestland | | Grassland | | Water body | | Building land | | Unutilized land | |
| --- | --- | --- | --- | --- | --- | --- | --- | --- | --- | --- | --- | --- |
|  | After  revision | Costanza et al. | After revision | Costanza et al. | After revision | Costanza et al. | After revision | Costanza et al. | After revision | Costanza et al. | After revision | Costanza et al. |
| Gas regulation | 4567.01 | *** | 1043.89 | *** | 652.43 | 49.7*** | 0 | 944.3*** | 0 | *** | 0 | *** |
| Climate regulation | 3523.12 | *** | 1174.37 | 1001.1*** | 1161.33 | *** | 600.24 | *** | 0 | *** | 0 | *** |
| Water conservation | 4175.55 | *** | 1043.89 | 14.2*** | 782.92 | *** | 26593.05 | 32226.9*** | 0 | *** | 39.15 | *** |
| Waste treatment | 1709.37 | *** | 1709.37 | 617.7*** | 2139.97 | 617.7*** | 23722.36 | 29656.7*** | -9799.5 | *** | 13.05 | *** |
| Soil formation and protection | 5088.95 | *** | 2544.48 | 71*** | 1905.1 | 7.1*** | 13.05 | *** | -3209.96 | *** | 26.1 | *** |
| Biodiversity conservation | 4253.84 | 170.4*** | 1422.29 | 14.2*** | 926.45 | 163.3*** | 3249.1 | *** | 0 | *** | 443.65 | *** |
| Food production | 130.49 | 383.4*** | 391.46 | 305.2*** | 1304.86 | 475.7*** | 130.49 | 1881.5*** | 0 | *** | 13.05 | *** |
| Raw material | 3392.64 | *** | 65.24 | 979.8*** | 130.49 | *** | 13.05 | 752.6*** | 0 | *** | 0 | *** |
| Recreational culture | 1670.22 | *** | 52.19 | 482.8*** | 13.049 | 14.2*** | 5663.09 | 10330.5*** | 0 | *** | 13.05 | *** |

*Note: * representative to make changes*
