# Supplementary material for: Spatio-temporal variation of ecosystem services value in the Northern Tianshan Mountain Economic zone from 1980 to 2030
Source: PeerJ. 2020 Aug 5;8:e9582. doi: 10.7717/peerj.9582 (PMC7414770; doi:10.7717/peerj.9582)
Supplement: Supplemental Information 4 [file peerj-08-9582-s004.docx]

**Table S4 Meteorological station information**

| x | y | DEM | Name |
| --- | --- | --- | --- |
| 89.12 | 42.56 | 34.50 | Tulufan |
| 82.54 | 44.37 | 320.10 | Jinghe |
| 82.34 | 45.11 | 336.10 | Alashankou |
| 84.51 | 45.37 | 449.50 | Kelamayi |
| 86.87 | 47.70 | 471.00 | Buerjin |
| 95.08 | 43.46 | 479.00 | Diaomaohu |
| 87.28 | 47.07 | 500.90 | Fuhai |
| 86.90 | 44.19 | 520.00 | Hutubi |
| 86.24 | 48.03 | 532.60 | Habahe |
| 82.05 | 44.85 | 533.00 | Bole |
| 83.00 | 46.44 | 534.90 | Tacheng |
| 88.05 | 47.44 | 735.30 | Aletai |
| 93.31 | 42.49 | 737.20 | Hami |
| 89.31 | 46.59 | 807.50 | Fuyun |
| 87.62 | 43.83 | 836.00 | Wulumuqi |
| 87.42 | 40.38 | 846.00 | Tieganlike |
| 88.13 | 42.14 | 922.40 | Kumishi |
| 86.08 | 41.45 | 931.50 | Kuerle |
| 82.78 | 41.22 | 984.00 | Shaya |
| 81.16 | 40.33 | 1012.20 | Alaer |
| 86.34 | 42.05 | 1055.30 | Yanqi |
| 83.36 | 45.56 | 1077.80 | Tuoli |
| 88.19 | 43.21 | 1103.50 | Dabancheng |
| 80.14 | 41.10 | 1103.80 | Akesu |
| 79.03 | 40.30 | 1161.80 | Keping |
| 77.65 | 38.90 | 1179.00 | Maigaiti |
| 77.16 | 38.26 | 1231.20 | Shache |
| 85.33 | 38.09 | 1247.20 | Qiemo |
| 75.59 | 39.28 | 1289.40 | Kashi |
| 85.43 | 46.47 | 1291.60 | Hoboksar |
| 76.17 | 39.72 | 1313.00 | Atushi |
| 79.56 | 37.08 | 1375.00 | Hetian |
| 78.17 | 37.37 | 1375.40 | Pishan |
| 82.43 | 37.04 | 1409.50 | Minfeng |
| 81.39 | 36.51 | 1422.00 | Yutian |
| 94.40 | 41.32 | 1573.80 | Hongliuhe |
| 93.03 | 43.36 | 1677.20 | Balitang |
| 94.42 | 43.16 | 1728.60 | Yiwu |
| 88.11 | 43.91 | 1815.00 | Tianchi |
| 75.15 | 39.43 | 2175.70 | Wuqia |
